# Evaluation of Short-Term Symptoms Associated With COVID-19 Vaccines Used Among Adolescents in Saudi Arabia

**DOI:** 10.7759/cureus.29306

**Published:** 2022-09-18

**Authors:** Fares K Alrowdhan, Abdulnasser Alwably, Abdulaziz S Altala, Hassan Alfaraj, Mhd Noor Farawaty, Rakan S Shaheen, Suliman A Alenazi, Einas M Yousef

**Affiliations:** 1 Clinical Medical Sciences, College of Medicine, Dar Al Uloom University, Riyadh, SAU; 2 Basic Medical Sciences, College of Medicine, Dar Al Uloom University, Riyadh, SAU; 3 Histology and Cell Biology, Faculty of Medicine, Menoufia University, Shibin Elkom, EGY

**Keywords:** teenagers, side effects, pfizer-biontech vaccine, moderna mrna-1273 vaccine, covid-19

## Abstract

Objectives: Several government-sponsored reporting systems have stated mild to moderate side effects of COVID-19 vaccines. However, patient-reported data on COVID-19 vaccine-associated adverse effects in adolescents are lacking. Our objective was to assess the short-term side effects of Pfizer-BioNTech BNT162b2 or Moderna mRNA-1273 vaccinations among teenagers in Saudi Arabia.

Methods: A retrospective, cross-sectional study was conducted among individuals aged 12-18 years old who received one of the two mentioned vaccines between July 2021 and March 2022 in Riyadh, Saudi Arabia.

Results: The most common short-term side effects reported for COVID-19 vaccines among teenagers in our study were fatigue, pain at the site of injection, fever, chills, headache, nausea, and vomiting. Female participants, individuals who had a history of severe acute respiratory syndrome coronavirus 2 (SARS-CoV-2) infection, and those who received two doses of the vaccine are at higher risk to develop side effects after getting the vaccine. Importantly, asthmatic participants have a higher incidence of COVID-19 vaccine side effects when compared to those with no history of chronic diseases.

Conclusion: Our findings might enhance public trust in the COVID-19 vaccine, which could speed up the immunization procedure.

## Introduction

On March 2020, World Health Organization declared the severe acute respiratory syndrome coronavirus 2 (SARS-CoV-2) outbreak in China a global pandemic. Most countries implement unprecedented preventive measures and precautionary strategies to reduce the burden of COVID-19 till herd immunity is achieved. Vaccination against COVID-19 is considered a crucial measure to help end the COVID-19 pandemic [1]. Several studies to develop a COVID-19 vaccine were immediately launched in the hopes of containing this pandemic. On December 11, 2020, the Pfizer-BioNTech mRNA vaccine (BNT162b2) was licensed by the Food and Drug Administration (FDA) for use in individuals aged ≥16 years old [2]. Furthermore, on May 10, 2021, the FDA approved the Pfizer-BioNTech vaccine for teenagers aged 12-15 years [3]. Only the Pfizer-BioNTech vaccine was approved for adolescents aged 12 to 17 in the United States as of July 30, 2021 [4]. Later on, a second mRNA vaccine Moderna mRNA-1273 was filed for emergency use authorization from the FDA in adolescents aged 12 to 17 in the United States [5].

The Kingdom of Saudi Arabia (KSA) has commenced an early vaccination campaign as a continuation of its outstanding efforts and actions to control the spread of the SARS-CoV-2 virus. The first vaccines licensed for adult immunization in KSA were the Pfizer-BioNTech vaccine (BNT162b2) and the Oxford-AstraZeneca vaccine (ChAdOx1 nCoV-19). On June 27, 2021, The Saudi Food and Drug Authority (SFDA) authorized the Pfizer-BioNTech vaccine (BNT162b2) for adolescents aged 12 to 18. Currently, both Pfizer-BioNTech (BNT162b2) and Moderna (mRNA-1273) COVID-19 vaccines are now registered in the KSA for those aged 12-17 years and older [6].

Many government-sponsored reporting systems have documented mild to moderate side effects and reactions to COVID-19 vaccines following the first or second doses of different types of COVID-19 vaccines. These symptoms include redness or swelling at the site of injection, pain, fever, muscle pain, fatigue, nausea, headache, vomiting, chills, and joint pain. Although adverse events related to vaccination are uncommon and usually mild or moderate, some studies reported new-onset neurologic symptoms [7], vaccine-induced thrombotic thrombocytopenia, cerebral venous sinus thrombosis [8], and some allergic reactions [9] following the vaccination of adults. In addition, perimyocarditis has been reported following the COVID-19 vaccination of adolescents [10]. Although many countries started vaccinating children between the ages of 12 and 18, the literature review showed a lack of patient-reported data on the COVID-19 vaccine's related symptoms in this age range.

The current study aimed to evaluate the short-term side effects of Pfizer-BioNTech BNT162b2 or Moderna mRNA-1273 vaccines among adolescents using an online survey in Riyadh, KSA. We used data from 1,080 individuals, aged 12-18 years, who received the Pfizer-BioNTech BNT162b2 or Moderna mRNA-1273 vaccines between July 2021 and March 2022. Our results will be beneficial for the public, clinicians, and healthcare professionals in comprehending and being aware of the potential implications of coronavirus immunization based on patients’ reports.

## Materials and methods

Study design and setting

A retrospective, cross-sectional study was conducted to evaluate the short-term post-vaccination symptoms and side effects of different types of COVID-19 vaccines in individuals aged 12 and 18 years old. The study included those who receive vaccinations from July 2021 to March 2022, in Riyadh, KSA. Data collection was initiated after obtaining the Institutional Review Board (IRB) approval from the College of Medicine, Dar Al Uloom University, KSA (IRB No.: Pro21110005). The study was conducted as per the ethical guidelines of the institutional and national research committees and the Declaration of Helsinki.

Study population and sample size

Our inclusion criteria were as follows: both male and female participants, aged 12-18 years, received either single or double doses of Pfizer-BioNTech BNT162b2 or Moderna mRNA-1273 vaccines in Riyadh, KSA. The study excluded individuals >18 years old and those who are <12 years old. The sample size is calculated using raosoft.com (http://www.raosoft.com/samplesize.html) [11] when we considered the population aged 10-19 years old in the KSA (around five million), and the sample size was calculated to be 385 when considering a 95% level of significance. However, to increase the statistical power of our study and reduce the sampling bias, we tried to increase the number of participants.

Data collection tool

The data were collected using a web-based, anonymous, self-completion questionnaire that was validated in a previous study after taking the permission of the corresponding author [12]. This questionnaire is available in both Arabic and English and built using Google Forms (American Association of Public Opinion Research's (AAPOR) Transparency Initiative, California, United States). Participants received the survey through personal email and social media. We also used direct contact with the researchers’ network using personal emails of teaching staff and employees in different universities in Riyadh, KSA. The survey begins by obtaining parental consent. The study questionnaire is composed of two main sections. The first section collected data about gender, age, chronic conditions, and SARS-CoV-2 infection status of the participants. The second section collects data about the COVID-19 vaccine such as the type of received vaccine, date of administration, first or second doses, list of side effects commonly reported in the literature, side effects timing, and duration. An open-ended question was included at the end of the questionnaire to enquire about any side effects that were not included in our survey and our participants may have experience. Doctors’ visits, hospital admission, and used medications after receiving the vaccine were also included in the questionnaire.

Statistical analysis

R Software version 3.5.2 (R Foundation for Statistical Computing, Vienna, Austria) "Eggshell Igloo" was used for all statistical analyses. Descriptive analysis for quantitative data includes mean and standard deviation (SD), for normally distributed variables. When normal distribution was violated, the median and interquartile ranges were used. For qualitative categorical variables, count and percentage were applied. A comparative analysis of the incidence of post-vaccination side effects across subjects with and without a history of COVID-19 infection was conducted. Univariate and multivariate logistic regression models were used to evaluate the adverse effects associated with different types of COVID-19 vaccines among study participants who experienced post-vaccination side effects taking into consideration participants' age, gender, chronic diseases, and history of COVID-19 infection (crude and adjusted odds ratio). p values <0.05 were considered statistically significant.

## Results

General characteristics of the participants

This cross-sectional, retrospective study was conducted in Riyadh, KSA, between July 2021 and March 2022 among 1,080 COVID-19 vaccine recipients aged 12-18 years. Among the included participants, 98.2% (No. = 1012) completed the questionnaire. Table 1 provides an overview of the general characteristics of the participants in the current study.

**Table 1:** Descriptive analysis of study participants’ general characteristics

Participants’ characteristics		Count (%) (No. = 1,012)
Age (years)	12-15	322 (31.8)
	16-18	690 (68.2)
Gender	Male	742 (73.6)
	Female	266 (26.4)
Chronic diseases	No	852 (84.2)
	Asthma	57 (5.6)
	Asthma and obesity	7 (0.7)
	Diabetes	14 (1.4)
	Diabetes and asthma	1 (0.1)
	Diabetes, asthma, and obesity	1 (0.1)
	Hypertension	9 (0.9)
	Hypertension and asthma	1 (0.1)
	Obesity	44 (4.3)
	Others	26 (2.6)
Have you got infected with COVID-19 after/before receiving the vaccine?	No	653 (64.5)
	Yes, before the vaccine	152 (15)
	Yes, after the vaccine	207 (20.5)
Did you receive two doses?	No	27 (2.7)
	Yes	985 (97.3)
Type of received vaccine	Pfizer	926 (91.5)
	One dose Pfizer, one dose Moderna	71 (7.0)
	Moderna	15 (1.5)
Do you have side effects?	No	455 (45.1)
	Yes	554 (54.9)

We next compared the general characteristics of those who experienced COVID-19 vaccine adverse effects to those who did not. A statistically significant difference was detected between these two groups regarding the type of received COVID-19 vaccine (p = 0.028). We also detected that more than 72% of participants who did not experience COVID-19 vaccination side effects did not have a history of SARS-CoV-2 infection, which was statistically significant in comparison to participants who reported side effects (p < 0.001) (Table 2).

**Table 2:** Comparative analysis of general characteristics among participants with and without COVID-19 vaccination side effects

Participants’ characteristics		Participants without side effects (No. = 455)	Participants with side effects (No. = 554)	p value
Age	12-15	155 (34.1)	166 (30.0)	0.175
	16-18	300 (65.9)	388 (70.0)	
Gender	Male	325 (71.4)	415 (75.5)	0.15
	Female	130 (28.6)	135 (24.5)	
Presence of health conditions		187 (12.5%)	102 (18.4)	0.25
Type of received vaccine	Pfizer	428 (94.1)	495 (89.4)	0.028
	One dose Pfizer, one dose Moderna	22 (4.8)	49 (8.8)	
	Moderna	5 (1.1)	10 (1.8)	
History of COVID-19 infection	No	331 (72.7)	320 (57.8)	<0.001
	Yes, before the vaccine	46 (10.1)	105 (19.0)	
	Yes, after the vaccine	78 (17.1)	129 (23.3)	

Side effects reported after getting the COVID-19 vaccine

**Figure 1:**
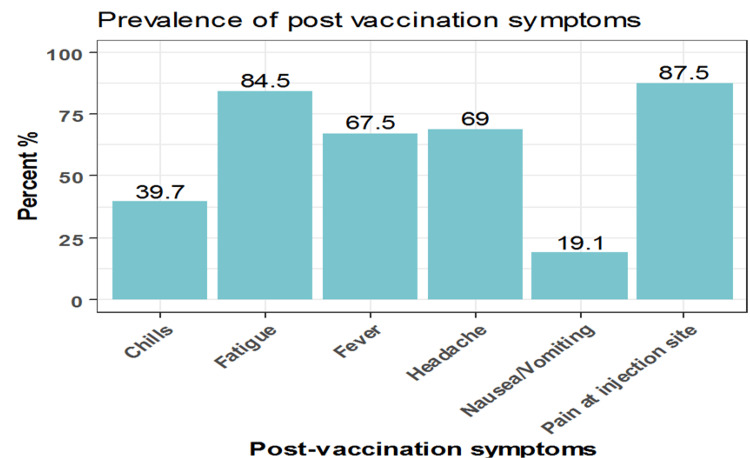
Prevalence of COVID-19 vaccination adverse effects

Among participants who experienced COVID-19 vaccination adverse reactions (54.9%, No.= 554), 87.5% had pain at the site of injection, 84.5% reported fatigue, 69% had a headache, 67.5% had a fever, 39.7% had chills, and 19.1% had nausea and vomiting (Figure 1). Other adverse effects had been recorded by the participants such as menstrual disturbance, lymph node enlargement, muscle and bone aches, runny nose, red eye, flu, and drowsiness. Of note, 75.6% of the participants reported using medications to avoid or mitigate vaccination side effects; however, about 9% of those participants had to visit the doctor after the onset of symptoms, and only 2.3% needed admission to the hospital because of the symptoms associated with the received vaccine. The vaccine side effects lasted from one to two days in 67.1%, three to five days in 25.8%, and more than five days in 7.0% of the participants (Table 3).

**Table 3:** How participants deal with COVID-19 vaccination side effects

Variable	Count (%) (No. = 554)
Have you used medications to mitigate side effects?	
No	135 (24.4)
Yes	419 (75.6)
Did you have to visit the doctor after the onset of symptoms?	
No	505 (91.2)
Yes	49 (8.8)
Have you been admitted to the hospital because of the symptoms associated with the vaccine?	
No	541 (97.7)
Yes	13 (2.3)
How long do side effects last?	
From one to two days	372 (67.1)
From three to five days	143 (25.8)
More than five days	39 (7.0)

Effect of gender and age on COVID-19 vaccination side effects

The univariate analysis of the association between gender and the occurrence of COVID-19 vaccination adverse effects revealed that female participants have a significantly higher incidence of fatigue, pain at the site of injection, fever, and nausea and vomiting than males (OR: 2.00, 2.07, 1.66, and 1.89, respectively) (Table 4). Although female participants were more likely to experience chills and headaches, no significant difference was detected. The increased risk of fatigue, pain at the site of injection, and nausea and vomiting in females when compared to males remained significant (OR: 1.93, 2.36, and 1.74, respectively) after adjusting for participants' age, chronic diseases, history of SARS-CoV-2 infection, and type of the received vaccine. Importantly, no significant association was detected between age groups and the occurrence of COVID-19 vaccination adverse effects.

**Table 4:** Univariate and multivariate analyses of the effect of gender on the risk of COVID-19 vaccination adverse effects OR and 95% CI of gender show a ratio of female to male. cOR: crude odds ratio (univariate analysis), aOR: adjusted odds ratio (multivariate analysis). * indicates p <0.05.

Side effects	Male (No.= 415)	Female (No.= 135)	cOR (95% CI)	aOR (95% CI)
	No. (%)	No. (%)		
Fatigue	342 (82.4)	122 (90.4)	2.00 (1.11-3.90*)	1.93 (1.04-3.83*)
Pain at the site of injection	356 (85.8)	125 (92.6)	2.07 (1.07-4.42*)	2.36 (1.16-5.34*)
Fever	270 (65.1)	102 (75.6)	1.66 (1.08-2.61*)	1.50 (0.96-2.39)
Chills	156 (37.6)	63 (46.7)	1.45 (0.98-2.15)	1.23 (0.82-1.86)
Headache	283 (68.2)	97 (71.9)	1.19 (0.78-1.84)	1.16 (0.74-1.84)
Nausea and vomiting	69 (16.6)	37 (27.4)	1.89 (1.19-2.98*)	1.74 (1.06-2.80*)

Effect of chronic diseases on COVID-19 vaccination side effects

Our univariate regression model demonstrated a significant increase in the incidence of fatigue (7.5 folds), chills (2.4 folds), and nausea and vomiting (2.2 folds) among asthmatic participants when compared to participants without a history of chronic diseases. After adjustment to the participants' age, gender, history of SARS-CoV-2 infection, and type of the received COVID-19 vaccine, the odds of fatigue and chills increased significantly by about 7 and 2.3 folds, respectively, among asthmatic participants when compared to participants who did not have a history of chronic disease. However, after adjustment, the odds of nausea and vomiting increased non-significantly by about 2 folds among asthmatic participants when compared to participants without a history of chronic disease (Table 5). In addition, none of the adverse effects were significantly more likely to occur among participants with other chronic diseases such as diabetes, hypertension, and obesity when compared to participants without a history of chronic diseases (Appendix 1, Table 6).

**Table 5:** Univariate and multivariate analyses of the effect of asthma on the incidence of COVID-19 vaccination adverse effects The reference is the participants with no chronic diseases. cOR: crude odd ratio, aOR: adjusted odd ratio, * indicates p <0.05.

Side effects	Univariate analysis cOR (95% CI)	Multivariate analysis aOR (95% CI)
Fatigue	7.53 (1.59-134.72*)	6.98 (1.46-125.29*)
Pain at the site of injection	0.57 (0.25-1.48)	0.52 (0.22-1.3)
Fever	1.14 (0.56-2.47)	1.14 (0.55-2.50)
Chills	2.39 (1.21-4.84*)	2.29 (1.15-4.69*)
Headache	1.60 (0.74-3.85)	1.53 (0.70-3.72)
Nausea and vomiting	2.22 (1.04-4.55*)	2.00 (0.91-4.16)

Effect of the type and number of received doses of COVID-19 vaccine on the incidence of side effects

Overall, our results indicate a non-significant difference between the type of COVID-19 vaccine schedule and the incidence of adverse effects. Both univariate and multivariate analyses demonstrated that the incidence of all reported side effects (fatigue, pain at the site of injection, fever, chill, headache, and nausea and vomiting) was less common among participants who received Pfizer-BioNTech vaccine compared to those who received Moderna vaccine (crude OR: 1.30, 0.79, 0.47, 0.62, 0.50, 0.33, respectively) (adjusted OR: 0.92, 0.79, 0.41, 0.41, 0.27, 0.32, respectively) (p > 0.05, non-significant) (Appendix 1, Table 6).

Regarding the number of received doses, the incidence of headache increased significantly among participants who received two doses of the vaccine by about 4 folds, and after adjustment, the incidence increased significantly by about 5.6 folds when compared to participants who received a single dose (OR = 4.01, 95% CI: 1.19-15.48, p = 0.028; and OR = 5.63, 95% CI: 1.42-28.63, p = 0.020), respectively. Of note, all other side effects increased among participants who received two doses of the vaccine; however, they fail to reach a statistically significant difference when compared to the participants who did not receive the two doses.

Effect of previous SARS-CoV-2 infection on COVID-19 vaccination side effects

We further analyzed the association between post-vaccination side effects and history of COVID-19 infection. Collectively, our analysis revealed that the incidence of post-vaccination symptoms increased significantly among participants with a history of COVID-19 infection. For those who got SARS-CoV-2 infection before getting the vaccine, a 2.4-fold increase in reporting the incidence of post-vaccination symptoms (OR = 2.36, 95% CI: 1.63-3.47, p < 0.001) was detected when compared to participants with no history of SARS-CoV-2 infection. Additionally, a 71% increase in the incidence of the symptoms was detected among individuals with a history of COVID-19 infection after receiving the vaccination (OR = 1.71, 95% CI: 1.24-2.36, p = 0.001) as compared to persons without a history of SARS-CoV-2 infection. Our results indicated that the incidence of COVID-19 vaccination side effects is more common among individuals who have a history of SARS-CoV-2 infection.

We further conducted a multivariate regression analysis which demonstrated that the incidence of nausea and vomiting among participants who had COVID-19 infection after receiving the vaccine increased significantly by about 69% when compared to participants who did not have SARS-CoV-2 infection (OR = 1.69, 95% CI: 1.00-2.82, p = 0.047). Interestingly, our analysis detected a significant decrease in the incidence of pain at the site of injection in both univariate (48%) and multivariate analyses (46%) among participants with a history of SARS-CoV-2 infection after receiving the vaccine when compared to participants with no history of infection (OR = 0.52, 95% CI: 0.30-0.93, p = 0.024; and OR= 0.54, 95% CI: 0.30-0.98, p = 0.038), respectively.

## Discussion

In the present study, we aimed to assess the short-term side effects of Pfizer-BioNTech BNT162b2 or Moderna mRNA-1273 vaccines among adolescents (12-18 years old) in Riyadh, KSA. Our results demonstrated that 54.9% of the included participants in our study reported side effects following the COVID-19 vaccination. The most prevalent side effects after receiving the COVID-19 vaccines among the participants of the current study were pain at the site of injection (87.5%), fatigue (84.5%), headache (69%), and fever (67.5%). Our observation is consistent with the reports of previous studies and clinical trials [12-15]. Most participants in our study reported using medications to avoid or mitigate COVID-19 vaccination side effects, and about 9% had to visit the doctor after the onset of symptoms. This observation may be explained by the parents’ belief that anti-inflammatory drugs protect against the potential symptoms of the COVID-19 vaccine, and visiting the doctor is mainly for their assurance. Only 2.3% of the included teenagers needed admission to the hospital which indicates that fewer severe side effects were reported after receiving COVID-19 immunization. Consistent with a previous study, our results demonstrated that side effects lasted for a few days in most of the participants, and only around 7.0% of the study participants had symptoms for more than five days [12].

The present results demonstrated that the incidence of all studied COVID-19 vaccination adverse effects is higher in females than in males; however, fatigue, pain at the site of injection, fever, and nausea and vomiting only reach a statistical significance level. Our findings were in agreement with the results of previous studies on both teenagers and adults [12,15,16]. The sex difference in COVID-19 vaccine response in females can be explained by the stronger and faster innate and adaptive immune responses in females than in males leaving them more susceptible to a higher incidence of COVID-19 vaccination adverse effects [17-19]. Another possible explanation is that females carry two X chromosomes which are known to include several immune-related genes [20]. Lastly, hormonal variations can also modulate the immune response as estrogen tends to have an immunoenhancing effect and testosterone suppresses the immune system [21,22].

Consistent with previous studies and clinical trials, our results revealed that Pfizer-BioNTech is safer and associated with less incidence of side effects [16,23]. We detected that 94% of participants who did not experience post-vaccination adverse effects received the Pfizer-BioNTech vaccine. We further studied the effect of chronic diseases on the incidence of COVID-19 immunization side effects. A significant increase in the incidence of fatigue (7.5 folds), chills (2.4 folds), and nausea and vomiting (2.2 folds) among asthmatic participants was detected when compared to those with no history of chronic diseases. This is consistent with findings by Shi et al. that adults with asthma had a greater risk of COVID-19 hospitalization and should be prioritized for COVID-19 booster vaccinations [24]. More studies are needed to explore the potential impact of COVID-19 vaccines on asthmatic patients.

In the present study, our results demonstrated that the incidence of headaches increased significantly (5.6 folds) among participants who received two doses of the vaccine when compared to those who received a single dose. In alignment with our results, a previous meta-analysis study by Castaldo et al. reported a 2-fold increase in the risk of developing a headache after receiving the COVID-19 vaccine [25]. We also detected that the incidence of COVID-19 vaccination side effects is more common among individuals with previous exposure to SARS-CoV-2 compared to those with no history of infection. Besides, we detected that more than 72% of participants who did not experience COVID-19 vaccination side effects did not have a history of SARS-CoV-2 infection. Our findings are consistent with those of previous studies which demonstrate more side effects among individuals with a history of SARS-CoV-2 infection [16,26]. These findings could indicate that a single dose of the COVID-19 vaccine can be considered equal to a previous infection with SARS-CoV-2 in stimulating the immune system. It is probable that a greater incidence of headaches and post-vaccination side effects in those who have been previously exposed to SARS-CoV-2 is due to a systemic immune response to COVID-19 immunizations. However, more research needs to be conducted to confirm our findings.

One important limitation of our study is that we employed a self-administered online questionnaire to collect our data, which might be associated with response or self-reporting bias that can affect the reliability of the results. Additionally, data collection was conducted at any time after receiving the vaccine (immediately after receiving the vaccine for up to nine months), which could give more chances of underestimated, missed, or erroneous reporting of vaccine-related side effects. Finally, in this study, we only considered short-term COVID-19 vaccination side effects after receiving one or two doses. This can be justified by the fact that teenagers in Saudi Arabia were requested to receive a booster dose of a COVID-19 vaccine a few months after the beginning of our study.

## Conclusions

In conclusion, our findings clearly emphasized that COVID-19 vaccination is associated with mild tolerable and acceptable side effects which last for a short duration. These data confirm the consistent safety profile of COVID-19 immunizations that have been documented in earlier research and clinical trials, enhancing public trust in the vaccine. More research is needed to determine the safety of the COVID-19 vaccine after receiving the booster dose. Furthermore, the COVID-19 vaccines' medium- and long-term side effects should be evaluated.

## Appendices

Appendix 1

**Table 6:** Univariate and multivariate logistic regression models to evaluate the short-term post-vaccination symptoms associated with different types of COVID-19 vaccines * indicates <0.05; ** indicates <0.001. Multivariate analysis took into consideration participants' age, gender, chronic diseases, and history of COVID-19 infection. cOR: crude odds ratio, aOR: adjusted odds ratio, Inf: infinity, NA: not available.

Predictors		Fatigue		Pain at the site of injection		Fever		Chills		Headache		Nausea and vomiting	
		cOR (95% CI)	aOR (95% CI)	cOR (95% CI)	aOR (95% CI)	cOR (95% CI)	aOR (95% CI)	cOR (95% CI)	aOR (95% CI)	cOR (95% CI)	aOR (95% CI)	cOR (95% CI)	aOR (95% CI)
Age (years)	12-15	-	-	-	-	-	-	-	-	-	-	-	-
	16-18	1.23 (0.75-1.99)	1.14 (0.68-1.90)	1.20 (0.69-2.03, p = 0.514)	1.26 (0.71-2.19, p = 0.425)	1.31 (0.89-1.92)	1.25 (0.84-1.86)	0.96 (0.66-1.40)	0.92 (0.62-1.35)	1.19 (0.81-1.76)	1.15 (0.76-1.72)	0.68 (0.44-1.07)	0.64 (0.40-1.02)
Gender	Male	-	-	-	-	-	-	-	-	-	-	-	-
	Female	2.00 (1.11-3.90*)	1.93 (1.04-3.83*)	2.07 (1.07-4.42, p = 0.042)	2.36 (1.16-5.34, p = 0.025)	1.66 (1.08-2.61*)	1.50 (0.96-2.39)	1.45 (0.98-2.15)	1.23 (0.82-1.86)	1.19 (0.78-1.84)	1.16 (0.74-1.84)	1.89 (1.19-2.98**)	1.74 (1.06-2.80*)
Chronic diseases	No	-	-	-	-	-	-	-	-	-	-	-	-
	Asthma	7.53 (1.59-134.72*)	6.98 (1.46-125.29)	0.57 (0.25-1.48, p = 0.212)	0.52 (0.22-1.38, p = 0.159)	1.14 (0.56-2.47)	1.14 (0.55-2.50)	2.39 (1.21-4.84*)	2.29 (1.15-4.69*)	1.60 (0.74-3.85)	1.53 (0.70-3.72)	2.22 (1.04-4.55*)	2.00 (0.91-4.16)
	Asthma and obesity	Inf (0.00-NA)	Inf (0.00-NA)	0.55 (0.08-10.95, p = 0.601)	0.58 (0.08-11.56, p = 0.631)	2.01 (0.29-39.44)	2.33 (0.34-45.92)	0.00 (NA-Inf)	0.00 (NA-Inf)	0.69 (0.11-5.26)	0.74 (0.12-5.66)	0.00 (NA-Inf)	0.00 (NA-Inf)
	Diabetes	Inf (0.00-NA)	Inf (0.00-NA)	1.39 (0.26-25.67, p = 0.758)	1.13 (0.20-21.23, p = 0.913)	2.26 (0.57-14.93)	2.03 (0.49-13.76)	2.99 (0.89-11.54)	2.62 (0.77-10.27)	1.22 (0.35-5.64)	1.02 (0.28-4.79)	2.54 (0.65-8.61)	2.33 (0.58-8.26)
	Diabetes and asthma	Inf (0.00-NA)	Inf (0.00-NA)	0.00 (NA-Inf, p = 0.985)	0.00 (NA-Inf, p = 0.984)	Inf (0.00-NA)	Inf (0.00-NA)	Inf (0.00-NA)	Inf (0.00-NA)	0.00 (NA-Inf)	0.00 (NA-Inf)	0.00 (NA-Inf)	0.00 (NA-Inf)
	Hypertension	1.08 (0.17-20.75)	0.77 (0.12-15.24)	0.69 (0.11-13.40, p = 0.740)	0.70 (0.10-14.20, p = 0.758)	2.51 (0.40-48.28)	2.39 (0.37-46.75)	1.71 (0.31-9.31)	1.79 (0.32-9.98)	0.46 (0.08-2.50)	0.42 (0.07-2.32)	0.89 (0.05-5.61)	0.76 (0.04-4.99)
	Hypertension and asthma	Inf (0.00-NA)	Inf (0.00-NA)	Inf (0.00-NA, p = 0.989)	Inf (0.00-NA, p = 0.990)	Inf (0.00-NA)	Inf (0.00-NA)	Inf (0.00-NA)	Inf (0.00-NA)	Inf (0.00-NA)	Inf (0.00-NA)	Inf (0.00-NA)	Inf (0.00-NA)
	Obesity	2.69 (0.78-16.92)	2.56 (0.74-16.22)	1.73 (0.50-10.95, p = 0.463)	1.71 (0.48-10.91, p = 0.480)	0.85 (0.39-1.98)	0.85 (0.38-1.99)	1.58 (0.72-3.47)	1.56 (0.70-3.43)	0.67 (0.30-1.51)	0.64 (0.29-1.47)	0.56 (0.13-1.64)	0.51 (0.12-1.53)
	Others	1.40 (0.38-9.05)	1.34 (0.35-8.77)	1.94 (0.38-35.49, p = 0.526)	2.14 (0.41-39.65, p = 0.470)	1.38 (0.46-5.04)	1.44 (0.47-5.35)	1.49 (0.52-4.23)	1.50 (0.51-4.30)	2.98 (0.81-19.20)	3.02 (0.81-19.60)	0.68 (0.11-2.54)	0.60 (0.09-2.27)
History of COVID-19 infection	No	-	-	-	-	-	-	-	-	-	-	-	-
	Yes after vaccine	1.31 (0.74-2.42)	1.37 (0.76-2.58)	0.52 (0.30-0.93, p = 0.024*)	0.54 (0.30-0.98, p = 0.038*)	0.84 (0.54-1.29)	0.84 (0.54-1.31)	1.01 (0.66-1.54)	1.01 (0.65-1.55)	1.21 (0.78-1.91)	1.25 (0.79-1.99)	1.56 (0.94-2.55)	1.69 (1.00-2.82*)
	Yes before vaccine	1.10 (0.61-2.08)	1.05 (0.56-2.07)	1.02 (0.51-2.17, p = 0.966)	1.21 (0.58-2.75, p = 0.626)	0.86 (0.54-1.38)	0.77 (0.47-1.28)	1.30 (0.83-2.03)	1.26 (0.78-2.02)	1.22 (0.76-2.00)	1.24 (0.74-2.12)	1.23 (0.69-2.13)	1.09 (0.59-1.95)
Receive two doses	No	-	-	-	-	-	-	-	-	-	-	-	-
	Yes	3.21 (0.83-10.89)	2.74 (0.63-10.93)	1.58 (0.24-6.29, p = 0.564)	1.99 (0.27-9.89, p = 0.436)	1.75 (0.50-5.89)	1.62 (0.42-6.39)	3.02 (0.77-19.92)	4.05 (0.91-29.78)	4.01 (1.19-15.48*)	5.63 (1.42-28.63*)	1.07 (0.27-7.06)	2.03 (0.42-16.06)
Type of vaccine	Moderna	-	-	-	-	-	-	-	-	-	-	-	-
	One dose Pfizer, one dose Moderna	2.81 (0.35-17.19)	1.87 (0.21-12.72)	0.67 (0.03-4.46, p = 0.720)	0.49 (0.02-3.84, p = 0.554)	1.50 (0.20-7.71)	1.46 (0.18-8.38)	1.13 (0.28-4.55)	0.74 (0.15-3.27)	1.79 (0.23-9.56)	0.84 (0.09-5.36)	0.54 (0.13-2.40)	0.51 (0.11-2.51)
	Pfizer	1.30 (0.19-5.29)	0.92 (0.12-4.24)	0.79 (0.04-4.31, p = 0.825)	0.79 (0.04-5.08, p = 0.835)	0.47 (0.07-1.92)	0.41 (0.06-1.77)	0.62 (0.17-2.25)	0.41 (0.10-1.66)	0.50 (0.08-2.04)	0.27 (0.03-1.32)	0.33 (0.09-1.31)	0.32 (0.08-1.40)
